# Antenatal Diagnosis and Management of a Sacrococcygeal Teratoma in a Monochorionic Diamniotic Twin Pregnancy: A Case Report

**DOI:** 10.7759/cureus.93056

**Published:** 2025-09-23

**Authors:** Hugo Barros, Sara Paiva, Joana Augusto, Carla Duarte, Conceição Brito

**Affiliations:** 1 Department of Obstetrics and Gynaecology, Unidade Local de Saúde do Alto Ave, Guimarães, PRT; 2 Department of Obstetrics and Gynaecology, Unidade Local de Saúde Gaia e Espinho, Vila Nova de Gaia, PRT

**Keywords:** fetal magnetic resonance imaging, multidisciplinary management, prenatal diagnosis, sacrococcygeal teratoma, twin pregnancy

## Abstract

We present a case of a sacrococcygeal teratoma (SCT) diagnosed antenatally in one fetus of a monochorionic diamniotic twin pregnancy. A 39-year-old gravida with a history of hypothyroidism was referred at 12 weeks’ gestation, when monochorionicity was confirmed. A routine second-trimester ultrasound revealed a small presacral cystic mass in Twin A, which progressively enlarged throughout pregnancy. Fetal magnetic resonance imaging at 26 weeks confirmed a well-circumscribed presacral lesion without solid components, consistent with an Altman Type I SCT. Owing to progressive growth and the associated risk of rupture, delivery was achieved by cesarean section at 36+5 weeks. Postnatal examination confirmed a large sacral mass in Twin A, with normal neonatal adaptation to extrauterine life. Surgical resection is planned within the first month of life. This case underscores the importance of prenatal imaging and multidisciplinary perinatal management in optimizing outcomes for fetuses with SCT.

## Introduction

Sacrococcygeal teratomas (SCTs) are the most frequent congenital tumors, with an incidence of one in 20,000-40,000 live births [1], with a female predominance [2,3]. SCTs arise from pluripotent cells of all three germ layers: endoderm, mesoderm, and ectoderm. The molecular pathogenesis remains unclear, but abnormal migration and differentiation of primordial cells during early development are thought to contribute. These tumors typically develop along the midline, with the sacrococcygeal region being the most common site [3,4].

Most cases are sporadic and isolated, but early identification is crucial given the potential for serious perinatal complications. Prenatal diagnosis with ultrasound and fetal magnetic resonance imaging (MRI) enables close surveillance and facilitates delivery and neonatal care planning [2,3]. Ultrasound is key for assessing tumor size, vascularity, and complications, while MRI adds precision by delineating intrapelvic extension and organ compression. There is a significant risk of tumor rupture, particularly during delivery and the immediate neonatal period. For this reason, the involvement of a multidisciplinary team is essential to ensure optimal perinatal management and improve overall outcomes [1,4]. Postnatal outcomes are generally favorable when lesions are identified and managed early.

This report describes the prenatal course, delivery, and immediate postnatal management of a fetus with SCT in a monochorionic diamniotic twin pregnancy.

## Case presentation

A 39-year-old gravida 2, para 1, with a history of hypothyroidism and one previous vaginal delivery, was referred to our obstetric unit after the diagnosis of a monochorionic diamniotic twin pregnancy. A first-trimester ultrasound at 12 weeks and five days confirmed monochorionicity and diamnionicity, with both fetuses demonstrating normal anatomy and a low estimated risk for aneuploidies (<1 in 1000 for each fetus).

**Figure 1:**
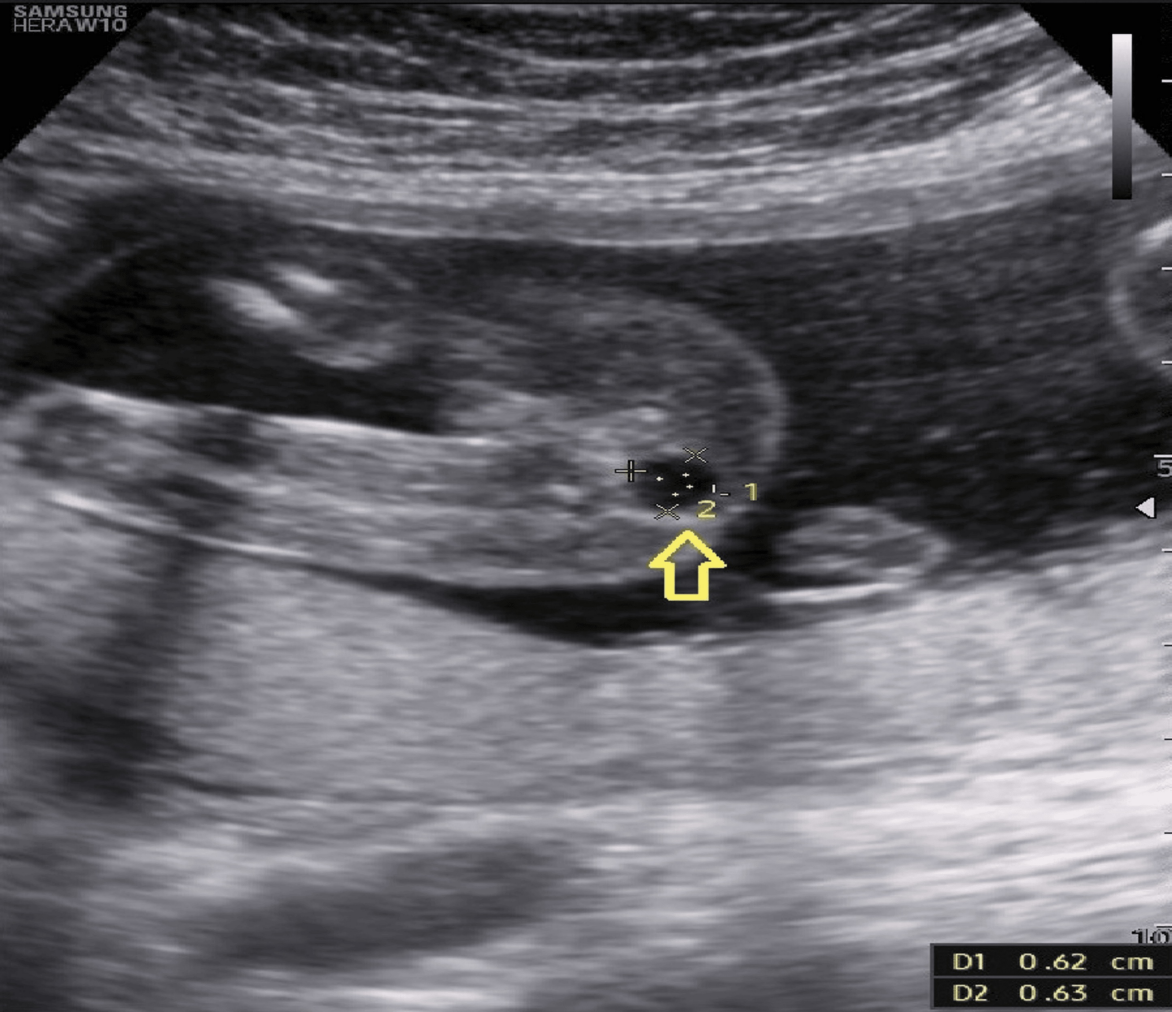
Ultrasound (transverse plane) at 21 weeks of the affected fetus showing a presacral cystic mass (arrow)

At 21 weeks, a routine second-trimester scan revealed a well-circumscribed presacral cystic lesion in Twin A, located on the maternal right side, measuring 7 × 6 × 6 mm (Figure 1). No additional structural abnormalities were detected in either fetus, and there were no sonographic signs of twin-to-twin transfusion syndrome or twin anemia-polycythemia sequence. Differential diagnoses included sacrococcygeal teratoma, anterior meningocele, enteric duplication cyst, and retrorectal tailgut cyst.

Serial ultrasounds performed every two weeks demonstrated progressive growth of the mass. Fetal Doppler studies remained normal, and no additional anomalies were identified. Invasive prenatal testing was offered but declined by the couple, as there were no other anatomical anomalies in either fetus, and amniotic fluid volumes were normal; therefore, in the absence of additional findings, they opted not to undergo invasive testing.

**Figure 2:**
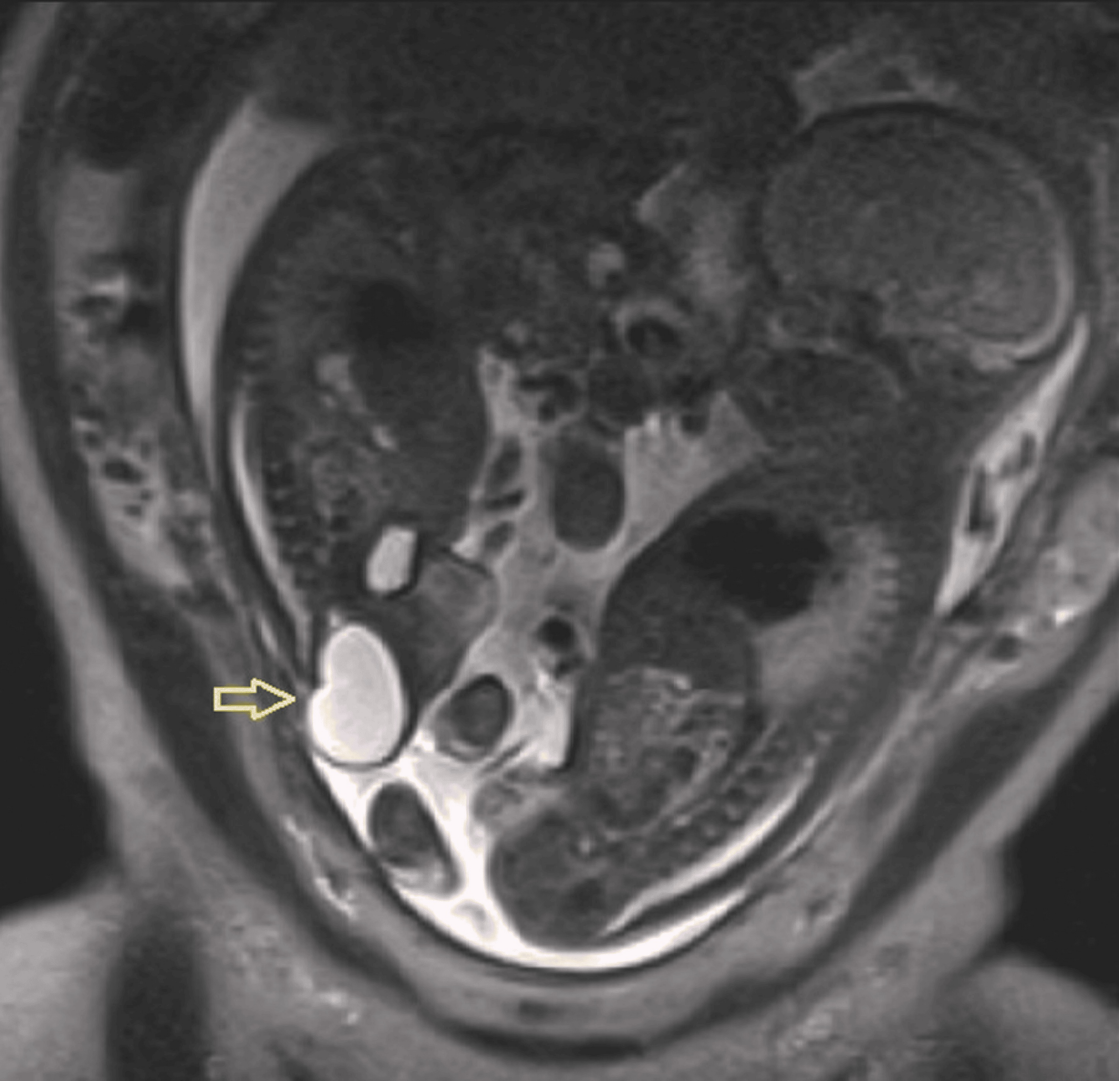
MRI (sagittal plane) at 26 weeks showing the cystic formation (arrow) in the affected fetus

A fetal echocardiogram at 24 weeks excluded cardiac anomalies. At 26 weeks, fetal MRI revealed a 35×25×29 mm presacral lesion, purely cystic on all sequences and without solid components, local invasion, spinal canal connection, or bone involvement, consistent with an Altman type I sacrococcygeal teratoma (Figure 2).

**Figure 3:**
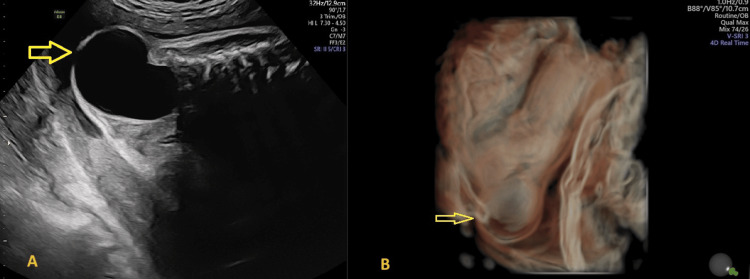
Ultrasound (sagittal view) at 35 weeks of (A) the progressively enlarging pelvic mass (arrrow), and (B) the corresponding 3D reconstruction (arrow) 3D: three-dimensional

By 35 weeks, the lesion in Twin A had enlarged to 51 × 48 × 42 mm, causing anterior displacement of the anus but without additional anatomical abnormalities, amniotic fluid imbalance, or hemodynamic compromise (Figure 3). Twin B remained structurally normal and appropriately grown. Given the progressive tumor growth and the risk of rupture during labor, an elective cesarean section was performed at 36 weeks and five days.

**Figure 4:**
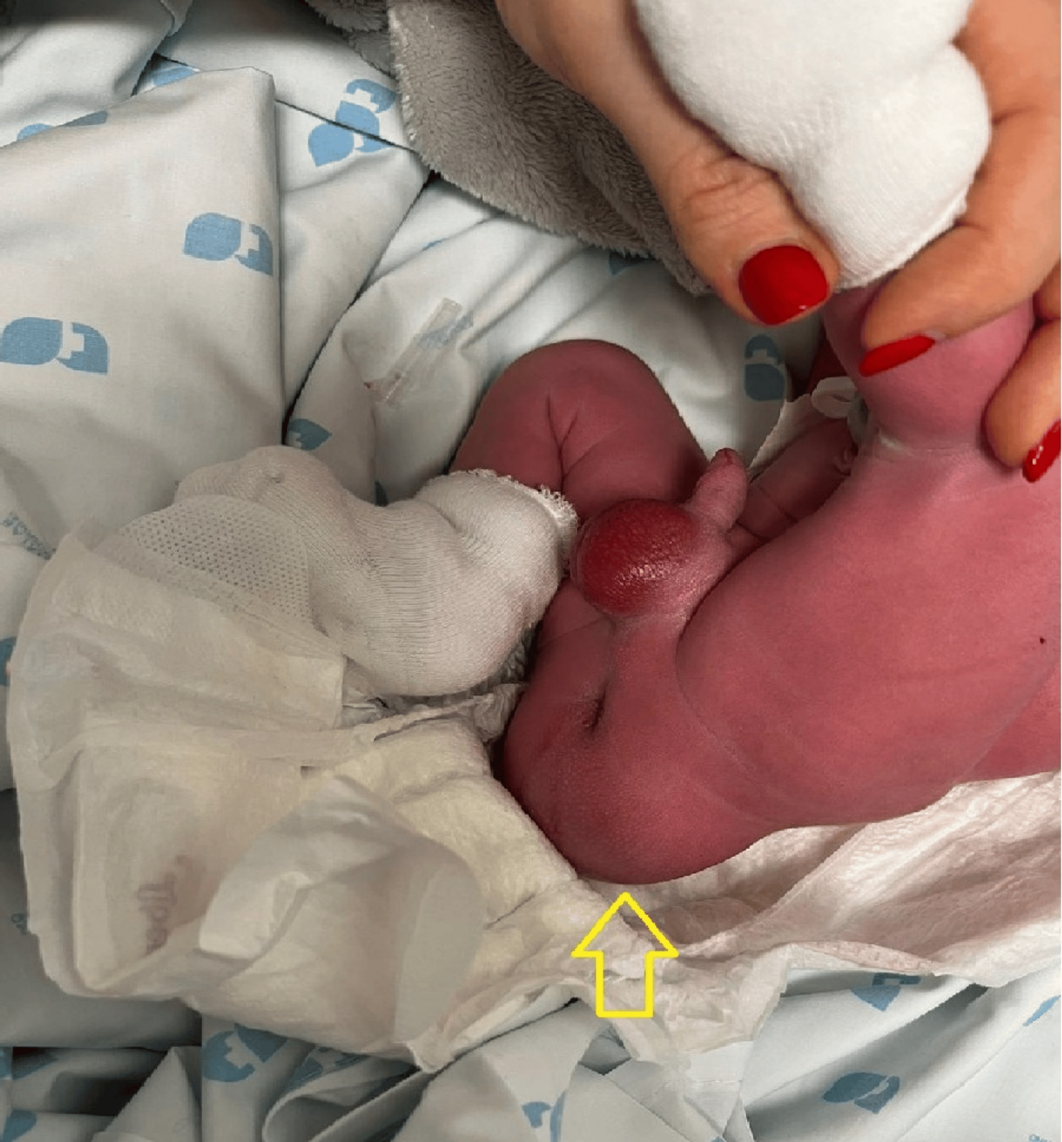
Image of the newborn post delivery showing a visible pelvic mass causing anterior displacement of the anus (arrow)

Twin A weighed 2620 g at birth, with Apgar scores of 9/10/10, and presented with a sacral mass measuring approximately 6 cm in diameter, causing deviation of the anus, which remained patent (Figure 4). Twin B weighed 2880 g, with Apgar scores of 9/10/10, and was phenotypically normal. The maternal postoperative course was uneventful, and both neonates adapted well to extrauterine life. The affected neonate was clinically stable, with normal micturition and passage of stool shortly after birth, and an otherwise unremarkable neonatal examination. Surgical resection is planned within the first month for definitive histopathological confirmation

## Discussion

SCTs are the most common congenital tumors, typically located in the presacral region. They occur in approximately one in 20,000-40,000 live births and represent the most frequent type of fetal teratoma [1]. A clear female predominance is noted, with around 80% of cases occurring in female fetuses [2,3].

SCTs are germ cell tumors containing at least two tissue types from the three embryonic germ layers, with variable differentiation. Although germ cell tumors can arise along the midline, the sacrococcygeal area accounts for 60-80% of cases [4]. The most widely accepted classification is the Altman system, developed by the American Academy of Pediatrics Surgical Section, which divides tumors into four types based on their internal and external components [2].

Most SCTs are identified during a second-trimester ultrasound, though some are diagnosed earlier. The majority of prenatal cases are Altman Type I or II [4]. Regular obstetric ultrasound surveillance, every two to three weeks, with Doppler assessment, is crucial. Changes in the structure of the cystic lesion, such as the development of solid components or increased vascularity, may impact the affected fetus as well as the co-twin in monochorionic pregnancies, highlighting the need for close monitoring of tumor growth, amniotic fluid volume, fetal cardiac function, and hemodynamic status, to detect complications such as anemia, hydrops, or cardiac failure, to promptly detect complications [3-5].

Fetal MRI improves delineation of SCTs, particularly in cases with suspected intrapelvic extension or organ compression, and is especially helpful when ultrasound findings are inconclusive [4]. Fetal echocardiography is essential for assessing cardiac function and guiding management decisions. Isolated SCTs are rarely associated with chromosomal abnormalities [4]. In the absence of additional anomalies, the risk of genetic alterations is low, and most fetuses have a normal karyotype. Therefore, invasive testing is generally not indicated unless other malformations or risk factors are present.

SCT management requires a multidisciplinary approach, including maternal-fetal medicine, obstetrics, radiology, and pediatric surgery. These tumors carry a significant risk of rupture during delivery and the early neonatal period. Vaginal delivery may result in traumatic hemorrhage, which can be life-threatening if not anticipated and promptly managed. Accordingly, the mode of delivery should be carefully planned, with cesarean section often preferred for large or highly vascularized tumors to minimize perinatal complications and optimize outcomes [1,3].

An elective cesarean section was scheduled during the 36th week of gestation following a multidisciplinary decision involving the Obstetrics, Neonatology, and Pediatric Surgery teams, due to the risk of spontaneous labor and the theoretical risk of cystic mass rupture during delivery, which could potentially result in an adverse fetal outcome.

Definitive treatment consists of complete surgical excision, including the coccyx, which is crucial to reduce recurrence, reported in 10-15% of cases [1,4,5], particularly with incomplete resection or malignant histology. Tumors with pelvic or intra-abdominal extension pose surgical challenges and increase the risk of pelvic nerve injury, potentially resulting in long-term urinary or fecal incontinence.

In select high-risk cases, such as fetuses with progressive hydrops, high-output cardiac failure, rapid tumor growth, or organ compression, prenatal interventions like open fetal surgery, laser or radiofrequency ablation, or serial amnioreduction may be considered to optimize perinatal outcomes [1,3,4].

Long-term follow-up after SCT resection is essential to monitor for tumor recurrence and potential bowel or bladder dysfunction, especially in patients with higher Altman types or prenatal evidence of obstruction. Recommended surveillance for three to five years includes clinical examinations every three to six months, monthly tumor markers (e.g., alpha-fetoprotein, lactate dehydrogenase), and periodic imaging of the primary site and chest. This approach facilitates early detection of recurrence or functional complications and supports ongoing patient management [1,3].

## Conclusions

This case illustrates the importance of detailed prenatal imaging in diagnosing and monitoring fetal SCTs, particularly in the context of multiple gestations, in a male fetus, which is rare for this type of teratoma. Early detection and multidisciplinary coordination enabled timely delivery and planning for postnatal surgery. In monochorionic twin gestations, early detection and multidisciplinary planning are indispensable to balance fetal surveillance and co-twin outcomes. Close surveillance and surgical intervention remain essential to ensure optimal outcomes.
